# Mir-20a-5p induced WTX deficiency promotes gastric cancer progressions through regulating PI3K/AKT signaling pathway

**DOI:** 10.1186/s13046-020-01718-4

**Published:** 2020-10-08

**Authors:** Jian Li, Danli Ye, Peng Shen, Xiaorong Liu, Peirong Zhou, Guifang Zhu, Yangwei Xu, Yun Fu, Xuanqi Li, Jingbo Sun, Jia Xu, Qingling Zhang

**Affiliations:** 1 Department of Pathology, Guangdong Provincial People’s Hospital, Guangzhou, Guangdong Province 510000 People’s Republic of China; 2 Department of Pathology, School of Basic Medical Science, Southern Medical University, Guangzhou, Guangdong Province 510282 People’s Republic of China; 3 Department of Oncology, Nanfang Hospital, Southern Medical University, Guangzhou, Guangdong Province 510515 People’s Republic of China; 4 Department of Oncological Sciences, The Tisch Cancer Institute, Icahn School of Medicine at Mount Sinai, New York, NY 10029 USA

**Keywords:** Gastric cancer, WTX/AMER1, miR-20a-5p, PI3K/AKT

## Abstract

**Background:**

The X-linked gene WTX (also called AMER1) has been reported to function as a tumour suppressor gene in Wilms’ tumour. In our previous study, WTX expression was shown to be significantly reduced in gastric cancer (GC), but the function and mechanism associated with WTX loss had yet to be fully elucidated.

**Methods:**

WTX expression and clinical significance were father analyzed in GC and control normal gastric tissues, and validated in public databases. The candidate pathway which was regulated by WTX during GC progression was searched by KEGG pathway analysis. The miRNA which monitored WTX expression was screened by miRNA microarray. After verified the pathway and miRNA both in vitro and in vivo, the relationship of miRNA, WTX and the downstream pathway were analyzed by Western blot, immunohistochemistry, RT-PCR, Co-immunoprecipitation (Co-IP), and luciferase analyses.

**Results:**

The results showed that WTX serves as a tumour suppressor gene in GC. The loss of WTX which is associated with the aggressiveness of GC by promoting GC cell proliferation in vitro and high metastasis in vivo*.* Furthermore, WTX expression was positively correlated with the overall survival of GC patients. Microarray assays, bioinformatics analysis, and verification experiments showed that WTX loss activates the PI3K/AKT/mTOR pathway and promotes GC cell proliferation and invasion. And the aberrant miR-20a-5p upregulation contributes to WTX loss in GC, which stimulates PI3K phosphorylation to activate PI3K/AKT/mTOR signaling pathway and promoted GC progression.

**Conclusions:**

The results of the present study elucidated the mechanism of GC progression, which is at least partially caused by aberrant miR-20a-5p upregulation leading to the inhibition of WTX expression and PI3K/AKT/mTOR signaling pathway activation. These findings provide a comprehensive understanding of the action of the miR-20a-5p/WTX/PI3K/AKT/mTOR signaling pathway in the progression and metastasis of GC.

## Background

Gastric cancer (GC) is the fourth most common cancer, the third leading cause of cancer-associated mortality in males and the fifth leading cause of cancer-associated mortality in females worldwide. Almost half of the GC patients were Chinese, most of them were at an advanced stage with lymph node or distal metastasis when diagnosed [1, 2]. Although a number of GC treatments have been developed in recent years, the prognosis of GC patients remain poor [3]. Thus, it is important to elucidate the new mechanisms of GC development to identify new ways to prevent GC progression.

Wilms’ tumour gene on the X chromosome (WTX), also known as AMER1 or FAM123B, was the first tumour suppressor gene located on the X chromosome discovered in the Wilms’ tumour [4–6]. Because of its unique location, a “one hit” somatic inactivation that targets the single allele in males or the active X allele in females can cause WTX gene inactivation [7]. The loss of WTX is also associated with the tumorigenesis of nephroblastoma [8]. The results of mechanistic studies have shown that WTX inhibits the WNT/β-catenin signaling pathway activation by binding β-catenin [9]. Furthermore, WTX antagonizes WNT/beta-catenin signaling by promoting β-catenin ubiquitination and degradation [10]. WTX is also able to enhance p53 acetylation by interacting with CBP/p300 protein [11]. However, as a candidate tumour-suppressor gene, the expression and functions of WTX have not been fully elucidated in other tumours.

In our previous study, WTX was shown to be generally lost in GC, and the stomach was identified as another important target organ of the WTX gene [12]. The WTX gene has a 16.3% [13, 14] to 30% [15] mutation rate in Wilms’ tumour, which drives WTX lost. However, WTX mutations and high methylation levels of WTX promoters are rare among GC patients [16, 17], suggesting that there may be another mechanism responsible for WTX gene silencing in GC patients. Aberrant microRNA (miRNA) expression is another important factor involved in regulating gene silencing. miRNAs can regulate gene expression at the post-transcriptional level by binding to the 3′-untranslated region (3’UTR) of target mRNAs. Interestingly, miRNAs have been reported to regulate the transcription of approximately 60% of genes [18] that participate in several bioprocesses, including organ development, cell proliferation, apoptosis [19, 20], and EMT [21, 22]. However, whether the loss of WTX in GC is associated with miRNAs remains unknown.

The phosphoinositide-3 kinase (PI3K)–protein kinase (PKB/AKT)–mammalian target of rapamycin (mTOR) pathway plays a crucial role in regulating multiple cellular functions and inducing pathological processes, including the cell cycle, proliferation, quiescence, tumorigenesis, and progression [23–25]. Deregulation of PI3K/AKT/mTOR pathway has been frequently demonstrated in GC and is closely associated with GC tumorigenesis and prognosis [26–29]. However, a relationship between WTX and the PI3K/AKT/mTOR pathway has yet to be reported. Therefore, to better understand the function of WTX and identify new markers for targeted GC therapy, in the present study, in vitro and in vivo assays and bioinformatics analyses were performed to elucidate the function and mechanisms of WTX loss and the relationship between WTX and the PI3K/AKT/mTOR pathway in GC progression.

## Methods

### Tissue specimens and cell culture

One hundred sixty-one cases of GC and matched adjacent normal gastric tissue samples were obtained from patients at Nanfang Hospital, Southern Medical University. No chemotherapy or radiation therapy was administered prior surgery. Prior patient consent and approval were obtained from the Institutional Research Ethics Committee. GC cell lines (GES-1, SGC7901, AGS, BGC803, MGC803, MGC823, BGC823, MKN28, and MKN45) were obtained from the Cell Bank of Chinese Academy of Medical Science (Shanghai, China). These cells were cultured in PPMI-1640 medium containing 10% fetal bovine serum (Invitrogen Life Technology) and incubated at 37 °C under a humidified atmosphere with 5% CO_2_. All cell lines were routinely tested for mycoplasma, the results of which were negative.

### Overexpression and knockdown cell lines

A lentivirus expression vector (LV-W-Puro) harbouring the entire WTX CDS region was synthesized by Invitrogen Co. (Shanghai, China). WTX shRNAs fragments were synthesized by Genechem Co. (Shanghai, China), verified and used to establish lentivirus expression vector (LV-shW-Puro). MiR-20a-5p mimics and inhibitor lentiviruses (LV-20 am-puro and LV-20ai-puro) were constructed by GenePhama (Shanghai, china). Target cells (2 × 10^5^) were infected with 1 × 10^6^ lentivirus transducing units in the presence of polybrene (1 μg/ml) and subsequently selected with puromycin (2 μg/ml) for ~ 5 days to establish the stable overexpression and knockdown cell lines.

### Cell proliferation, colony formation, migration, and wound healing assays

Cell proliferation was assessed using the cell counting kit-8 (CCK8) assay. Briefly, 2 × 10^3^ cells were seeded into each well of 96-well plates in triplicate. After incubating for the indicated time, 5 μl of CCK-8 buffer (DOJINDO, Japan) was added to each well, and the cells were incubated for 1 h at 37 °C. Then, the absorbance of each well was measured at 450 nm using a Microplate Autoreader (Bio-Rad, Hercules, CA, USA).

For colony formation assays, cells were plated in 6-well plates (300 cells/well) and cultured for approximately 2 weeks until the appearance of cell colonies. The colonies were fixed and stained with hematoxylin, and those containing more than 50 cells were counted for further statistical analysis. Each experiment was performed thrice.

For cell migration assay, 1 × 10^5^ cells were seeded into Transwell chambers (BD Biosciences, San Jose, CA, USA) under the same treatment conditions described above and incubated for 48–72 h. Cells that had migrated through the filter were stained with haematoxylin and counted in five fields per insert for further statistical analysis.

For wound healing assays, pretreated cells were seeded into 6-well plates and scratched with a pipette tip after 24 h. Then, after the abraded, floating cells were removed with PBS, the adhered cells were continuously cultured for another 48 h, with images taken every 8 h. Each experiment was performed thrice.

### Immunohistochemistry (IHC), in situ hybridization (ISH), and scoring

Paraffin-embedded sections were dewaxed from xylene, rehydrated in a graded ethanol series to water, and used for IHC staining according to a previously described protocol [12]. The samples were incubated with primary antibodies against the following proteins: WTX (diluted 1:200, Abcam, Cambridge, UK), Ki-67 (diluted 1:500, Abcam, Cambridge, UK), p-AKT and p-mTOR (diluted 1:200, Cell Signaling Technology, Danvers, MA).

ISH assay were performed under RNase-free conditions. The miR-20-5p probe and ISH kit were purchased from Boster Co. (Wuhan, China). The slides were stepwise dewaxed, rehydrated in a graded ethanol series to water, and refixed following a previously described protocol [12]. Then, they were incubated in 50 μg/ml Proteinase K, prehybridize for 4 h at 60 °C, 1.5 μg/ml probe incubated 18 h at 60 °C in series; following rinsing 2×, 1×, and 0.1 × SSC; blocking, anti-digoxin-biotin, SABC-POD, streptavidin-HRP, DAB substation, and hematoxylin counterstain in sequence to detect the positive signal.

The results were scored as previously described as a sum of the staining intensity and percentage of positive tumour cells [12]. Briefly, the staining intensity was scaled from 0 to 3. The percentage of cells showing positive staining was scored from 0 to 4. The final staining score (0–12) was calculated as the sum of the intensity and percentage scores and was then adapted to 4-point IRS scores as follows: 0–1 (−), 2–3 (+), 4–8 (+ +) and 9–12 (+ + +). Finally, we set (+) as the WTX expression cut off point, with (−) and (+) ~ (+ + +) indicating negative and positive expression, respectively. The clinical pathological significance of the IHC data were analyzed by Chi-square analysis. IHC staining and scoring were performed in a blind manner.

### Immunofluorescence (IF)

The cells were cultured in dishes and then fixed with 4% paraformaldehyde at 4 °C for 15 min. Then, after being blocked with normal goat serum for 30 min, the cells were incubated with primary antibodies against WTX (1:200) and PI3K (1200) at 4 °C overnight, after which they were incubated with Alexa Fluor 488-conjugated Affinipure goat Anti-mouse IgG (H + L) and 594-conjugated goat anti-rabbit IgG(H + L) (Proteintech, Chicago, IL) for 2 h at room temperature. Subsequently, DAPI was used to stain cell nuclei. Then, the stained cells were mounted with 80% glycerol/PBS for subsequent examination by a laser-scanning confocal microscope (FV1000, Olympus, Japan) using FV10-ASW 4.0 viewer software (Olympus, Japan).

### Immunoblotting

The proteins were eluted with denaturation buffer; separated by SDS-PAGE, and transferred to PVDF membranes (Pierce Biotechnology, Rockford, IL, USA). Subsequently, the menbranes were blocked in milk before being incubated in primary antibodies (WTX, diluted 1:500, Abcam; PI3K, p-PI3K, AKT, p-AKT, mTOR, p-mTOR, P70S6K, 4E-BP1, diluted 1:1000, Cell Signaling Technology, Danvers, MA, USA; GAPDH, diluted 1:2000, Proteintech, Rosemont, IL, USA) and the secondary antibodies. Finally, the proteins were visualized using an enhanced chemiluminescence detection system (Amersham Biosciences Europe, Germany) according to the manufacturer’s instructions. All experiments were repeated at least three times.

### RNA extraction and quantitative reverse transcription PCR (RT-PCR)

Total RNA was extracted from tissues and cells lines with RNAiso-Plus (TAKARA, Dalian, China). Single-stranded cDNA was then synthesized from 1 μg extracted mRNA using RT-PCR cDNA synthesis kit (TAKARA, Dalian, China) according to the manufacturer’s instructions. RT-PCR was performed using an Applied Biosystems 7500 Sequence Detection system with iQ™ SYBR green supermix (Bio-Rad Laboratories, Hercules, CA, USA). Primers were prepared as described previously [12]. Thermal cycling conditions were as follows: 95 °C 10 min for 1 cycle; 95 °C 5 s, 60 °C 30 s, and 72 °C 34 s for 40 cycles followed by the melting curve stage. The relative expression of WTX and miR-20a-5p were evaluated based on the threshold cycle (Ct), which was calculated as 2^−ΔΔCT^. All of the samples were analysed in three independent experiments, each with four technical replicates performed in each assay.

### Subcutaneous and orthotopic xenograft tumour mouse model

Subcutaneous tumour models were established in 4-week-old BALB/c nude mice (nu/nu) (the Animal Center of Southern Medical University, Guangzhou, China) by subcutaneously injecting 100 μl of a cell suspension (1 × 10^6^ cells) into the right inguinal region of each nude mouse. The tumour volumes were measured every 2 days starting 10 days after injection. Tumour diameter was measured using a slide caliper, and the volume was calculated using the following formula: tumour volume (mm^3^) = (L × W^2^)/2 [30]. After 30 days of observation, the mice were sacrificed to harvest the tumours for farther analysis.

Orthotopic GC mouse models were established in 4-week-old BALB/c nude mice. After anesthetizing the mice with ketamine (70 μg/kg), a small incision was made in the abdomen and to reveal the stomach. Then, 100 μl of a cell suspension (1 × 10^6^ cells) was injected into the muscle tissue of the stomach. Then, the stomach position was reset, the wound was treated with penicillin, and the abdominal incision was closed. The model mice were sacrificed 2 months after surgery to collect the stomachs, and the tumours were measured and fixed for further histopathological study.

### MRNA array

Total RNA was extracted from the AGS.veh and AGS. W cells using Trizol reagent (Invitrogen, Carlsbad, CA, USA) according to the manufacturer’s instructions. Subsequently, the total RNA samples were digested with DNaseI, purified using an RNeasy Kit (Qiagen, Hilden, Germany), after which the quantity and quality of the samples were assessed. Then, the RNA was used for cDNA synthesis and the production of biotin-tagged cRNA with a GeneChip IVT Labelling kit (Affymetrix), and the fragmented cRNA with controls were hybridized to each GeneChip array according to the manufacturer’s instructions. Hybridization, data capture, and analysis were performed by CapitalBio Co. (Beijing, China). The Affymetrix Human Genome U133 Plus 2.0 Array (Santa Clara, CA, USA), which contains more than 54,000 probe sets covering more than 47,000 transcripts and variants representing more than 38,500 genes, was used for microarray analysis. The differentially expressed genes were identified using threshold values of ≥2- and ≤ − 2-fold change and an FDR significance level of < 5%. The differentially expressed gene profiles data for the AGS.veh and AGS. W cell lines were deposited on Gene Expression Omnibus (GEO) under the accession code GSE114353.

### MiRNA array

Total RNA from GC and matched gastric mucosa tissue was labelled with Hy3 using a miRCURY LNA miRNA Power Labelling kit (Exqion, USA). Human lung fibroblast (HLF) cells was used as controls. MiRNA microarrays (CCDTM-miRNA850-V4p1.4) were provided by the Infectious Disease and Immunogenetics section, Department of Transfusion Medicine, Clinical Center, National Institutes of Health, USA. MiRNA array hybridization and data analysis were performed following the manufacturer’s instructions and a previously described protocol [31]. The microRNA expression profile data were deposited on GEO under the accession code GSE94882.

### Dual-luciferase reporter system analysis

A WTX gene 3’UTR fragment harbouring the miR-20a-5p binding site was PCR amplified from genomic DNA. The sequences of primers used in the present study were previously described [12]. The WTX gene 3’UTR fragment was specifically mutated using a QuickChange Site-directed Mutagenesis kit (TOYOBO, Shanghai, China). The wild-type or mutated 3’UTR fragments were subcloned into the vector pGL3-control (Promega, Madison, MI, USA) to construct the vectors miR-20a-p-5p-GL3-WTX-3’UTR-WT and miR-20a-p-5p-GL3-WTX-3’UTR-Mut, respectively. Then, the vectors were cotransfected with miR-20a-5p into MGC823 or SGC7901 cells, which were subsequently harvested and analyzed for luciferase activity according to the manufacturer’s instructions. Three independent experiments were performed.

### KEGG pathway enrichment analysis

Kyoto Encyclopedia of Genes and Genomes (KEGG) pathway enrichment analyses of DEGs were performed using the web-based tool DAVID 6.8 (https://david.ncifcrf.gov). The results were visualized in a Bar plot by using *ggplot2* in the R software environment.

### Statistical analyses

Each experiment was performed at least 3 times. All data were presented as the means± standard deviation (SD). Statistical analyses were performed using Prism 5 (GraphPad Software, San Diego, CA, USA). Two-tailed Student’s *t*-test was used to assess the significance of differences between two groups. One-way ANOVA was used to assess the significance of differences among multiple groups. Chi-square test was used to estimate the correlation between WTX expression and clinicopathologic features. Survival curves were analyzed using the Kaplan-Meier method and assessed by log rank testing. *P*-values< 0.05 were considered to be statistically significant.

## Results

### WTX loss correlates with GC progression and poor prognosis

**Fig. 1:**
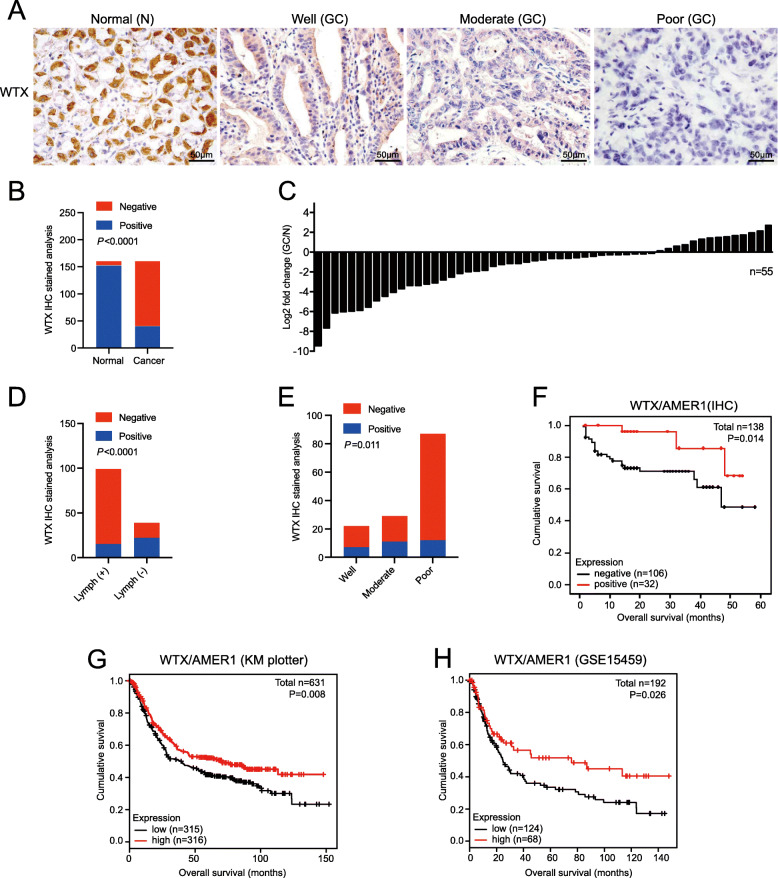
WTX loss correlates with advanced gastric cancer (GC) progression and poor patient prognosis. **a** IHC staining of WTX in normal (N) stomach mucosa and well-, moderately, and poorly differentiated GC tissues. Scale bars, 50 μm. **b** IHC staining analysis of WTX expression in GC and adjacent normal mucosal tissues. *n* = 161. Chi-square test. **c** Ratio (GC/N) of WTX mRNA expression in 55 GC tissues and adjacent normal mucosal tissues, as determined by RT-PCR. Expression levels were normalized to those of GAPDH. **d** IHC Staining analysis of WTX expression in GC tissues with or without lymph node metastasis. *n* = 138. Chi-square test. **e** IHC staining analysis of WTX expression in GC tissues with different differentiation characteristics. *n* = 138. Chi-square test. **f** Kaplan-Meier survival analysis of GC patients with positive or negative WTX expression based on IHC data. **g**-**h** Kaplan-Meier survival analyses of GC patients with high or low WTX expression based on KM plotter database and GSE15459 dataset

To investigate the role of WTX in gastric cancer (GC), we performed IHC staining of WTX using 161 cases of GC and matched adjacent normal gastric tissue samples (Fig. 1a). The level of WTX expression was significantly lower in GC than that in normal tissues (*P* < 0.001, Fig. 1b). RT-PCR results also confirmed that WTX mRNA expression was lower in 55 GC tissues than that in the matched adjacent normal mucosal tissues (Fig. 1c). In addition, positive WTX expression was observed for 15.2% (15/99) of groups with lymph node metastasis and 43.6% (17/39) of groups without lymph node metastasis (*P* < 0.001, Fig. 1d and Table 1), suggesting that WTX expression is negatively correlated with lymph node metastasis. Positive IHC staining of WTX was observed in 47.8% (11/23), 26.8% (15/56), and 13.4% (11/82) of well-, moderately, and poorly differentiated tumours, respectively (*P* = 0.011, Fig. 1e and Table 1), indicating that WTX expression is positively correlated with GC differentiation. Moreover, the loss of WTX expression was highly correlated with GC patient overall survival (*P* = 0.014, Fig. 1f and Table 1).

**Table 1:** Association of WTX expression with clinicopathological characteristics of gastric cancer

Clinicopathological variables	WTX expression (%)		*P*-value
	Negative	Positive	
*Age (years)*			
< 60	58 (79.5)	15 (20.5)	0.438
≥ 60	48 (73.8)	17 (26.2)	
*Gender*			
Male	72 (75.0)	24 (25.0)	0.447
Female	34 (81.0)	8 (19.0)	
*Tumor depth*			
T1	6 (85.7)	1 (14.3)	0.492
T2	20 (71.4)	8 (28.6)	
T3	73 (78.5)	20 (21.5)	
T4	6 (60.0)	4 (40.0)	
*Lymph node*			
-	22 (56.4)	17 (43.6)	0.000^∗^
+	84 (84.8)	15 (15.2)	
*Histological grade*			
Well	15 (68.2)	7 (31.8)	0.011^∗^
Moderate	18 (62.1)	11 (37.9)	
Poor	75 (86.1)	12 (13.9)	
*Stages*			
I	20 (77.0)	6 (23.0)	0.361
II	43 (70.5)	18 (29.5)	
III	35 (85.4)	6 (14.6)	
IV	7 (70.0)	3 (30.0)	
*Overall survival*			
Alive	76 (36.4)	31 (63.6)	0.006^∗^
Death	28 (90.3)	3 (9.7)	

∗ Statistically significant difference, 0.000: (*p* = 0.000365)

The relationship between WTX expression and the survival of GC patients was verified by an analysis of online GEO data with Kaplan-Meier plotter (www.kmplot.com) [32]. In the GEO, six datasets comprising 631 cases with demonstrated WTX expression are available, including GSE15459, GSE62254, GSE51105, GSE29272, GSE22377, and GSE14210. The results confirmed that compared to patients with high WTX expression, those with low WTX expression had a worse overall survival rate (*P* = 0.008, Fig. 1g). Clinicopathological data analysis based on the GSE15459 dataset showed that the overall survival rates in the low WTX expression group were significantly lower than those in the high WTX expression group (*P* = 0.026, Fig. 1h). The online data analysis results were also consistent with our IHC-based clinicopathological analysis. Taken together, these results suggested that the loss of WTX is associated with advanced GC progression, which could be used as a prognostic marker in GC. In summary, these results demonstrated that WTX loss is highly correlated with poor differentiation, lymph node metastasis, and poor prognosis in GC.

### WTX inhibits GC proliferation, migration, and invasion

**Fig. 2:**
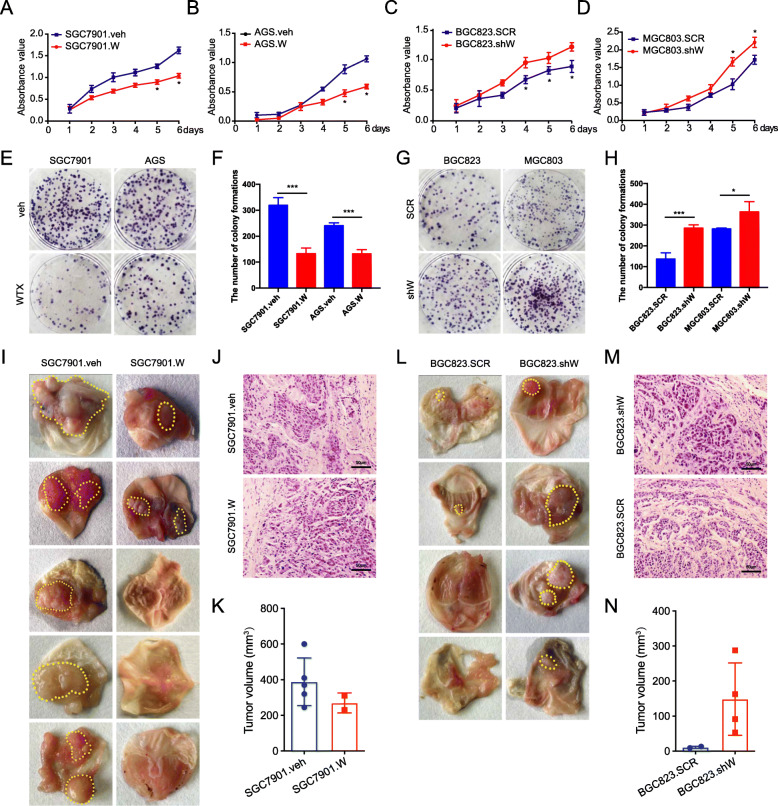
WTX negatively regulates GC proliferation. **a**-**d** Cell proliferation analyses of the indicated cells. *P < 0.05, means±SD, *n* = 3. Two-tailed Student’s *t*-test. **e**, **g** Representative images from colony formation assays for the indicated cells. **f**, **h** Quantification of colony formation for the indicated cells. ***P < 0.001, *P < 0.05, means±SD, *n* = 3. Two-tailed Student’s *t*-test. **i**, **l** Images of gross GC orthotopic tumours formed by the indicated cell lines. **j**, **m** Representative images of GC orthotopic tumours with H&E staining. Scale bars, 50 μm. **k**, **n** Volume analyses for the GC orthotopic tumours. means ± SD

WTX functions were further assessed in GC cell lines. Cells with low (SGC7901 and AGS) and high (BGC823 and MGC803) WTX expression were selected to establish WTX-overexpression (SGC7901.W and AGS.W) (Fig. S1a) and WTX knockdown (BGC823.shW and MGC803.shW) (Fig. S1b) cell lines, respectively. The cell counting kit-8 (CCK8) and colony formation assay results showed that the proliferation and colony formation ability of SGC7901.W and AGS. W cells was significantly inhibited compared to that of the controls, (Fig. 2a-b and e-f), while these abilities were significantly increased in MGC803.shW and BGC823.shW cells (Fig. 2c-d and g-h). Transwell and wound healing assay results showed that the invasion and migration abilities of the WTX overexpression cells were significantly lower than those of the control cells (Fig. S1c and e-f), whereas, they were improved in the WTX knockdown cells (Fig. S1d and g-h). These results indicate that WTX significantly inhibits the proliferation, migration, and invasion ability of GC cells.

Subcutaneous gastric tumour mouse models were established to analyse the effect of WTX on tumour formation and growth in vivo. The tumour take rate in both the control and SGC7901.W group were 100% (Fig. S1i). However, tumour growth was significantly inhibited in the SGC7901.W group compared to that observed in the control group (Fig. S1j-k). The orthotopic mouse tumour model observations showed that the tumour take rate were 100% (5/5) and 40% (2/5) in the SGC7901.veh and SGC7901.W groups, respectively (Fig. 2i-j). In addition, the average tumour volume in the SGC7901.W group was less than that of the SGC7901.veh group (Fig. 2k), and while liver metastasis occurred in SGC7901.veh orthotopic tumour model group, none was observed in the SGC7901.W group. The tumour take rate for the BGC823.SCR and BGC823.shW groups were 50% (2/4) and 75% (3/4), respectively (Fig. 2l-m), and the average tumour volume was generally higher in the BGC823.shW group than in the BGC823.SCR group (Fig. 2n). No metastasis was observed in the liver or the other organs. These results demonstrated that WTX could significantly inhibit tumour formation and metastasis in the orthotopic mouse tumour models, suggesting that WTX functions as a tumour suppressor in GC to inhibit GC cell proliferation, invasion and metastasis.

### WTX inhibits PI3K/AKT/mTOR pathway activation

**Fig. 3:**
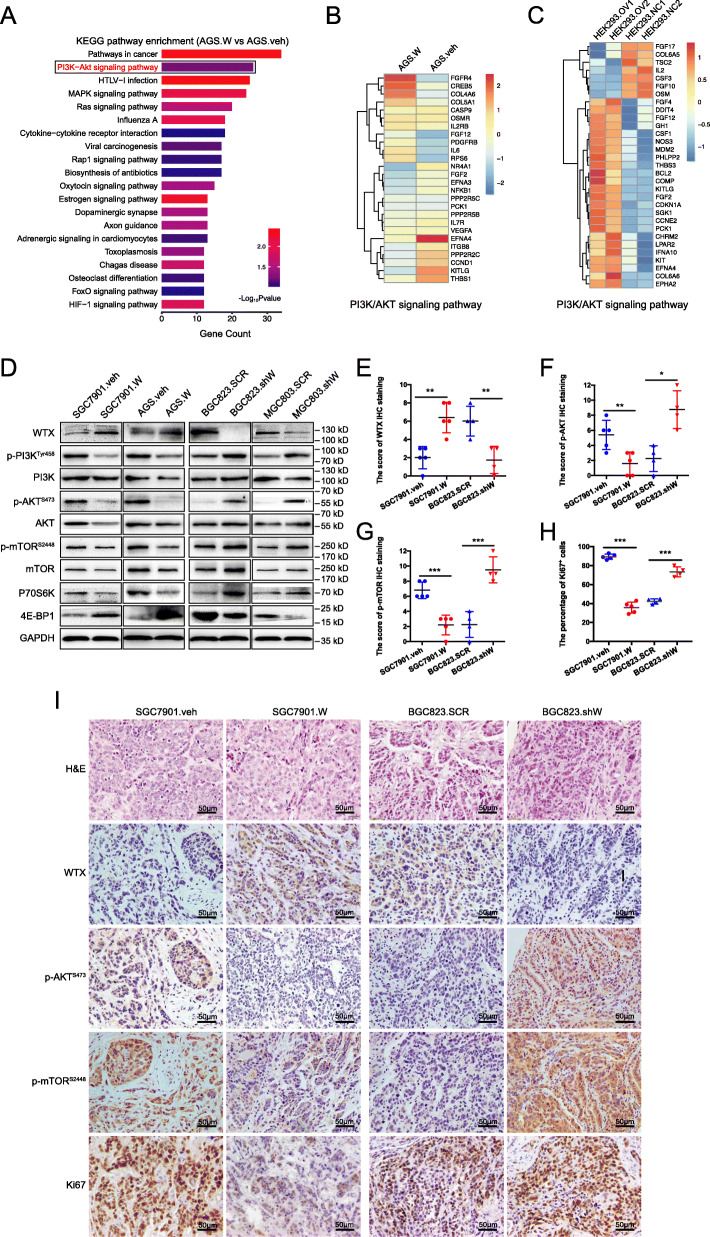
WTX inhibits PI3K/AKT/mTOR pathway activation. **a** The top 20 hits from the KEGG pathway enrichment analysis performed with microarray data for AGS. W and AGS.veh. **b** A heatmap based on 26 overlapping genes in PI3K/AKT signaling pathway identified from the AGS. W vs AGS.veh comparison. **c** A heatmap based on 31 overlapping genes in the PI3K/AKT signaling pathway in the GSE34715 dataset. **d** Western blot analyses of PI3K/AKT/mTOR pathway proteins in the indicated cells. GAPDH is shown as a loading control. **e**-**g** The WTX, p-AKT, and p-mTOR IHC staining scores in tumours of orthotopic models. ***P < 0.001, **P < 0.01, *P < 0.05, means±SD, *n* = 5. Two-tailed Student’s *t*-test. **h** The percentage of positively stained Ki-67 cells in tumours of orthotopic models. ***P < 0.001, **P < 0.01, *P < 0.05, means±SD, *n* = 5. Two-tailed Student’s *t*-test. **i** Representative images of H&E staining and WTX, p-AKT, p-mTOR, and Ki-67 IHC staining of tumours from orthotropic models

To elucidate the molecular mechanisms associated with how WTX inhibits GC cell proliferation and invasion, two KEGG pathway analysis were performed. Compared to other GC cell lines, WTX expression in AGS cells is relatively low, and its stable overexpression cell line (AGS.W) is more efficient. Therefore, the AGS. W and AGS.veh cell lines were used for gene expression profile studies to identify differential expressed genes (DEGs), which resulted in 617 upregulated and 628 downregulated genes being obtained. KEGG pathway analysis of these DEGs revealed that the PI3K/AKT pathway was dramatically inhibited in AGS. W cells (Fig. 3a~b and Fig. S2a). Interestingly, similar to our study, a similar study based on the GSE34715 dataset [11] also showed that PI3K/AKT signaling pathway was affected by WTX overexpression in HEK293 cells (Fig. 3c and Fig. S2b-c), suggesting that the PI3K/AKT pathway may be the major downstream signaling pathway of WTX in GC cells. Therefore, the total and phosphorylation levels of PI3K/AKT pathway proteins were analysed in WTX-overexpressing and knockdown cells. The results showed that compared to the control cells, the phosphorylation levels of PI3K, AKT, mTOR, and downstream signaling cascades were upregulated in both MGC803.shW and BGC823.shW cells but downregulated in SGC7901.W and AGS. W cells (Fig. 3d). These results indicated that WTX inhibits PI3K/AKT pathway activity in vitro. In addition, the levels of WTX, p-AKT, p-mTOR, and Ki-67 were also analysed by IHC staining in both the WTX-overexpressing and knockdown GC mouse tumour models described above. Comparing to the control groups, p-AKT and p-mTOR levels were reduced in WTX-overexpressing tumours but increased in WTX knockdown tumours (Fig. 3e-g and i). Ki-67 staining results showed that the proliferation index was significantly decreased in WTX-overexpressing tumours but increased in WTX knockdown tumours compared to that observed in the control groups (Fig. 3h-i). These results confirmed that WTX inhibits PI3K/AKT/mTOR pathway activity, promoting GC proliferation and invasion both in vitro and in vivo*.*

### Aberrant miR-20a-5p upregulation is associated with WTX loss in GC

**Fig. 4:**
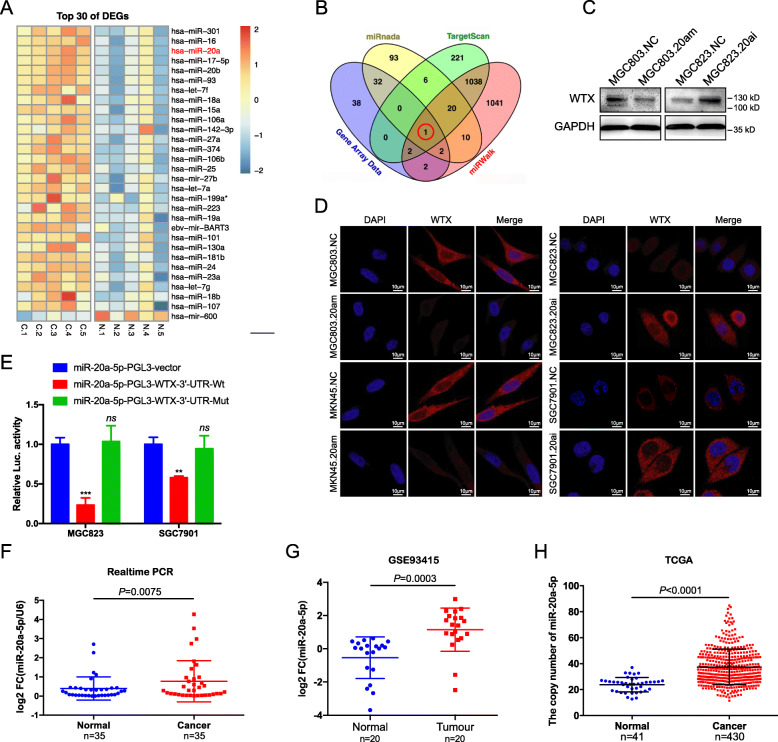
Aberrant miR-20a-5p upregulation leads to WTX loss in GC. **a** miRNA expression heatmap of GC and adjacent normal mucosal tissues based on the GSE94882 dataset. **b** The four-way Venn diagram reveals the numbers of overlapping miRNAs obtained using three publicly available bioinformatics algorithms (TargetScan, miRWalk and miRanda) and the microarray-based WTX signature. **c** Western blot analyses of WTX expression in the indicated cells. 20am = miR-20a-5p-mimics, 20ai = miR-20a-5p-inhibitor. GAPDH is shown as a loading control. **d** Immunofluorescence staining analysis of WTX expression and localization in the indicated cells. Scale bars, 10 μm. **e** Luciferase reporter assays were performed in MGC823 and SGC7901 cells cotransfected with Wt-3’UTR or Mut-3’UTR and miR-20a-5p mimics. ***P < 0.001, **P < 0.01, *ns* indicates the means are not significantly different (*P*>0.05), means±SD, *n* = 3. Two-tailed Student’s *t*-test. **f** RT-PCR analyses of miR-20a-5p expression in GC and adjacent normal mucosal tissues. means±SD. Two-tailed Student’s *t*-test. **g** MiR-20a-5p expression in GC based on GSE93415 dataset. means±SD. Two-tailed Student’s *t*-test. **h** MiR-20a-5p expression in GC based on TCGA. means±SD. Two-tailed Student’s *t*-test

The results of our previous studies have shown that the methylation levels of the WTX promoter are low in GC, and it was reported that WTX mutation is rare in GC patients [12, 16, 17], suggesting that some other mechanisms are responsible for WTX loss in GC. MiRNAs regulate the functions of many genes by targeting their mRNAs. To test if WTX expression is regulated by miRNAs in GC, we performed a miRNAs expression analysis on five human GC tissues with low WTX expression and matched adjacent normal gastric mucosa with normal WTX expression. The differentially expressed miRNAs were defined as having a minimum change in expression of greater than two-fold, and 77 upregulated miRNAs were identified (*P* < 0.01, Fig. 4a and Fig. S3a-b). The upregulated miRNAs were analysed using three published target prediction databases (*miRanda, TargetScan, and miRWalk*), and a Venn diagram was used to select the candidate miRNAs targeting WTX. The bioinformatics analysis of the microarray results suggested that miR-20a-5p is the top candidate regulator of WTX expression in GC (Fig. 4b).

Subsequently, we evaluated whether miR-20a-5p can regulate WTX expression and observed that miR-20a-5p overexpression inhibited WTX protein expression, while miR-20a-5p inhibition restored WTX protein expression (Fig. 4c). IF staining results also verified that miR-20a-5p negatively regulated WTX protein expression in the GC cell lines (Fig. 4d). Luciferase assay results confirmed that miR-20a-5p could directly bind to the WTX-3’UTR (Fig. S3c) in both MGC823 and SGC7901 GC cells (Fig. 4e). These data suggest that miR-20a-5p inhibits WTX expression by directly binding to the 3’UTR of WTX in GC cells.

To farther investigate the role of miR-20a-5p in regulating WTX, miR-20a-5p expression was assessed in both GC tissues and cell lines by RT-PCR. Significantly higher miR-20a-5p expression was observed in the GC tissues than in the adjacent normal gastric tissues (Fig. 4f). In addition, compared to the normal gastric mucosal cell line (GES-1), 6 of 8 GC cell lines exhibited high miR-20a-5p expression (Fig. S3d). Similarly, using a public database, an analysis of the GSE93415 set [33] showed that miR-20a-5p expression was significantly higher in GC tissues than in adjacent normal tissues (Fig. 4g and Fig. S3e-f). Furthermore, the GSE23739 dataset [34] and TCGA database analyses also confirmed that miR-20a-5p was more highly expressed in GC tissues than in normal control tissues (Fig. S3g-h and Fig. 4h). Our data together with other public data available online indicated that miR-20a-5p overexpression is significantly correlated with the aberrant loss of WTX in GC and functions by binding WTX.

### MiR-20a-5p promotes GC progress by inhibiting WTX to activate the PI3K/AKT/mTOR pathway

**Fig. 5:**
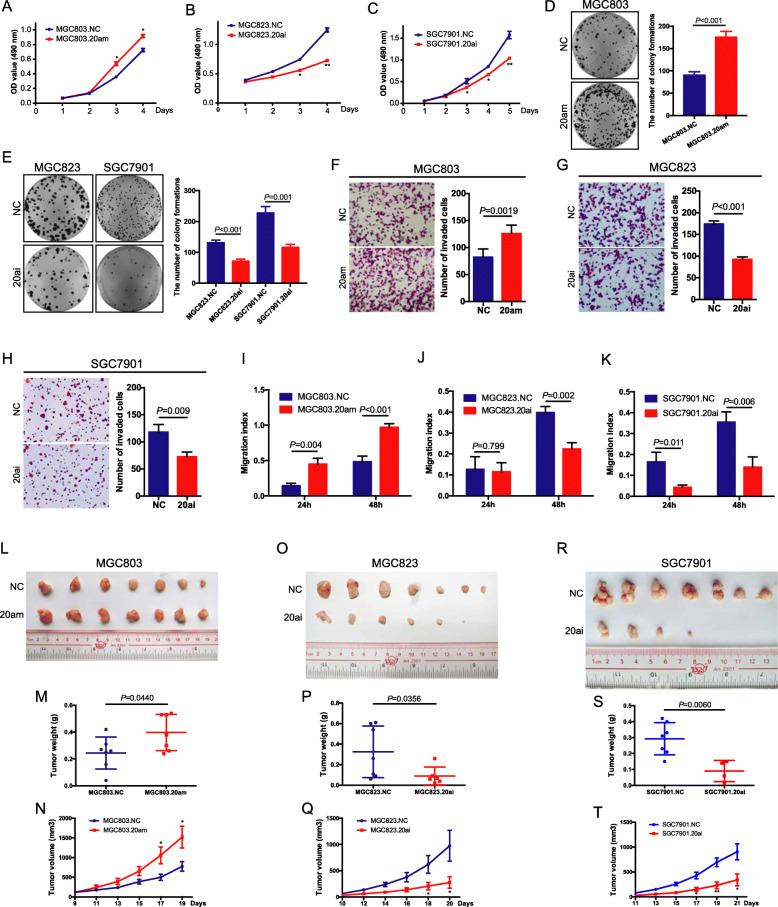
MiR-20a-5p promotes GC proliferation and migration. **a**-**c** Cell proliferation analyses in the indicated cells. **P < 0.01, *P < 0.05, means±SD, *n* = 3. Two-tailed Student’s *t*-test. **d**-**e** Colony formation analyses of the indicated cells. means±SD, *n* = 3. Two-tailed Student’s *t*-test. **f**-**h** Transwell cell migration analyses of the indicated cells. means±SD, *n* = 3. Two-tailed Student’s *t*-test. **i**-**k** Wound-healing analyses of the indicated cells. means±SD, *n* = 3. Two-tailed Student’s *t*-test. **l**, **o**, **r** Images of subcutaneous tumours formed by the indicated cells. *n* = 7. **m**, **p**, **s** Weight analyses of subcutaneous tumours formed by the indicated cells. means±SD, *n* = 7. Two-tailed Student’s *t*-test. **n**, **q**, **t** Growth curves of subcutaneous tumour formed by the indicated cells. **P < 0.01, *P < 0.05, means±SD, *n* = 7. Two-tailed Student’s *t*-test

**Fig. 6:**
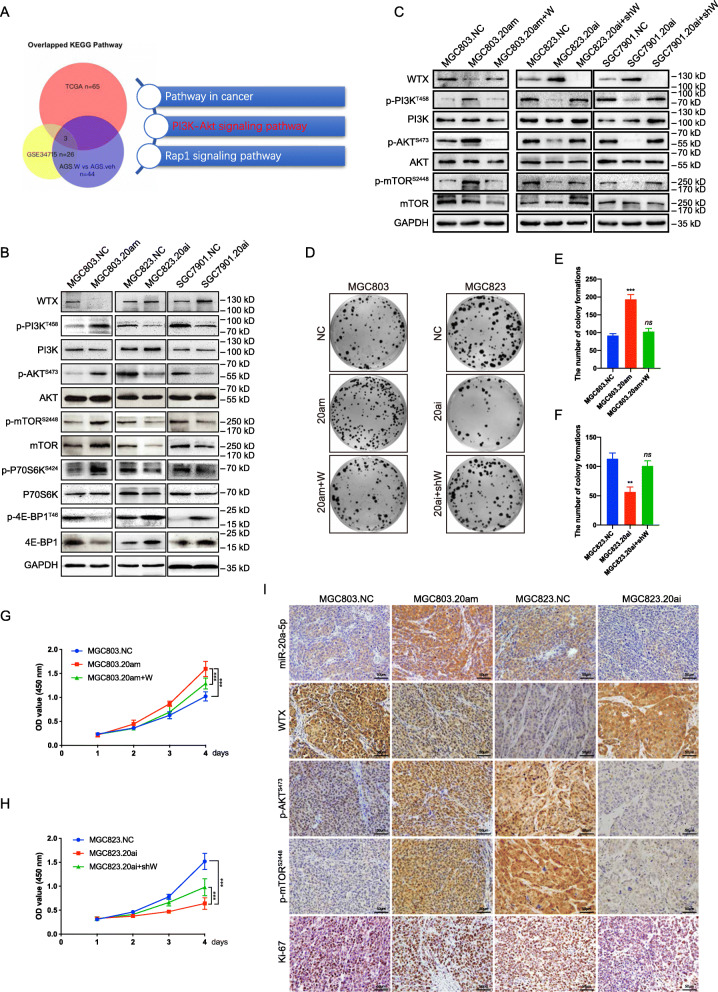
MiR-20a-5p regulates GC progression through the WTX/PI3K/AKT/mTOR pathway. **a** The three-way Venn diagram shows the number of overlapping pathway components in the KEGG pathway enrichment analysis of AGS. W *vs* AGS.veh (*n* = 44, Fig. 3a), the GSE34715 dataset (*n* = 26, Figure S2c), and TCGA (*n* = 65, Figure S5c). **b**-**c** Western blot analyses of PI3K/AKT/mTOR pathway proteins in the indicated cells. GAPDH is shown as a loading control. **d**-**f** Colony formation analyses of the indicated cells. ***P < 0.001, **P < 0.01, means±SD, *n* = 3. Two-tailed Student’s *t*-test. **g**-**h** Cell proliferation analyses of the indicated cells. ***P < 0.001, means±SD, *n =* 3. One-way ANOVA. **i** ISH staining of miR-20a-5p and IHC staining of WTX, p-AKT, and p-mTOR in subcutaneous tumours formed by the indicated cells. Scale bars, 50 μm

To further elucidate the biological functions of miR-20a-5p, MGC803 cells with low miR-20a-5p expression and relatively high WTX expression were used to establish an miR-20a-5p-overexpressing subclone cell line (MGC803.20 am). The MGC823 and SGC7901 cell lines, which exhibit relatively high miR-20a-5p and low WTX expression, respectively, were used to establish miR-20a-5p knockdown cell lines (MGC823.20ai and SGC7901.20ai) (Fig. S3d). MiR-20a-5p expression and WTX protein levels were verified by RT-PCR and Western blot analyses in these established cells (Fig. S4a-b and Fig. 6b). Compared to the control cells, MGC803.20 am cells showed significantly enhanced proliferation ability (Fig. 5a), colony formation (Fig. 5d), and migration capacity (Fig. 5f, i and Fig. S4c), while MGC823.20ai and SGC7901.20ai cells exhibited significantly inhibited proliferation (Fig. 5b~c), colony formation (Fig. 5e) and migration capacity (Fig. 5g-h, j-k and Fig. S4d).

Subcutaneous tumour models were established to assess tumour formation and growth in vivo, with the results showing that tumours grew faster in the MGC803.20 am group than in the MGC803.NC group (Fig. 5l-n). In addition, MGC823.20ai and SGC7901.20ai tumour models exhibited decreased tumour formation and slower growth than that observed in the control group (Fig. 5o-t). The Ki-67 index was higher in MGC803.20 am tumours and lower in SGC7901.20ai tumours than that observed in the controls (Fig. 6d and Fig. S4i-j). These in vitro and in vivo data showed that miR-20a-5p can promote the proliferation and migration of GC cells, indicating that miR-20a-5p may be important in inducing GC progression.

To elucidate the mechanism by which miR-20a-5p induces GC progression, we analysed miR-20a-5p expression and related pathways with the TCGA database. Forty-one cases with matched normal tissue data were selected from 428 GC cases in TCGA were included in our study (Fig. S3h). In 15 of 41 cases, miR-20a-5p expression was high in GC and low in matched normal tissues. Subsequently, we extracted the gene expression profile data for the 15 pairs GC and matched normal tissues from TCGA to perform DEG and KEGG pathway enrichment analysis (Fig. S5a), resulting in the identification of 4153 DEGs (*P* < 0.01, fold change>2, Fig. S5b) and 65 pathways associated with altered miR-20a-5p expression. One of the 65 pathways was the *PI3K/AKT signaling pathway* (Fig. S5c). To verified whether miR-20a-5p expression is consistently associated with the PI3K/AKT signaling pathway, we also performed KEGG pathway enrichment analyses using another two GC expression profile datasets, including our AGS. W vs AGS.veh dataset (GSE114353) and the public GEO dataset GSE34715. Interestingly, the PI3K/AKT signaling pathway was enriched out in all three assayed datasets (Fig. 6a), suggesting that the ability of miR-20a-5p to promote GC progression may involve the PI3K/AKT pathway.

Subsequently, we further analysed the changes in WTX and PI3K/AKT pathway component expression in miR-20a-5p-modified GC cells. Western blot results showed that WTX expression was inhibited and that the phosphorylation of PI3K, AKT, mTOR, and P70S6K was increased in MGC803.20 am cells, while WTX expression was increased and the phosphorylation of PI3K, AKT, mTOR, and P70S6K was decreased in MGC823.20ai and SGC7901.20ai cells compared to that observed in control cells (Fig. 6b). These results revealed that miR-20a-5p can inhibit WTX expression and activate the PI3K/AKT/mTOR pathway.

To elucidate the relationship and role of WTX in the process of miR-20a-5p-mediated activation of the PI3K/AKT/mTOR pathway, rescue assays were performed in MGC803.20 am cells, and WTX knockdown analysis was performed in MGC823.20ai and SGC7901.20ai cells. The Western blot results verified that the miR-20a-5p-mediated regulation of PI3K/AKT/mTOR pathway activation could be suppressed by WTX (Fig. 6c). In addition, the restoration of WTX in MGC803.20 am cells attenuated their enhanced proliferation resulting from miR-20a-5p overexpression, while WTX knockdown in MGC823.20ai cells rescued the proliferation of these cells that was suppressed by miR-20a-5p silencing (Fig. 6d-h).

**Fig. 7:**
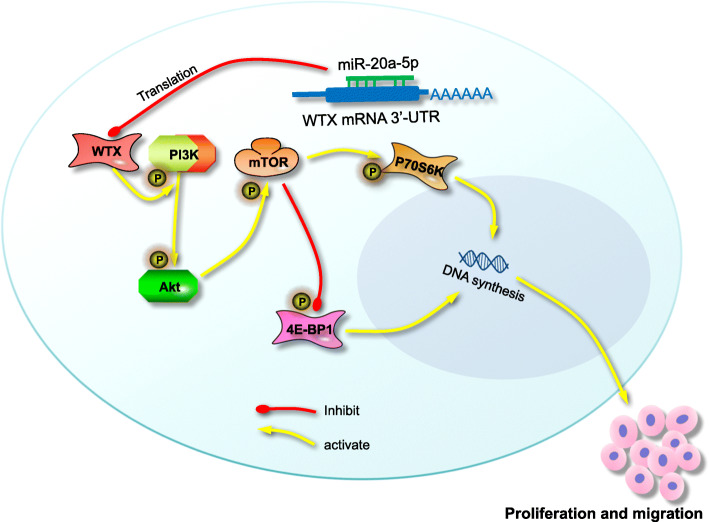
Model: WTX loss promotes GC proliferation and migration and is modulated by miR-20a-5p through the PI3K/AKT/mTOR pathway, ultimately leading to GC progression

The relationship among miR-20a-5p, WTX, and PI3K/AKT/mTOR pathway were farther verified in miR-20a-5p-modified subcutaneous GC tumours. IHC staining results demonstrated that miR-20a-5p could inhibit WTX expression and promote increased AKT and mTOR protein phosphorylation (Fig. 6i and Fig. S4e-h). Taken together, these results show that miR-20a-5p targeting of WTX promotes GC progression through the PI3K/AKT/mTOR pathway (Fig. 7).

## Discussion

Tumour suppressor genes are normal genes that slow down cell division, repair DNA mistakes, and contribute to the fidelity of the cell cycle replication process. The inactivation of tumour suppressor genes is an important contributor to GC development and progression [35]. In our previous study, WTX expression was shown to be generally lost in GC samples, and the stomach was identified as a novel target of WTX [12]. WTX plays a major role in both organ development and tumour suppression, but its functions have not been well elucidated [11]. The clinicopathological consequences of WTX loss in GC development were analysed, and the results showed that WTX loss is associated with the poor differentiation, high invasion, and proliferation phenotypes of GC, as well as being associated with increased lymph node metastasis and poor GC patient prognosis. These clinical data suggested that WTX loss is correlated with GC progression and can be used as a prognosis marker in GC patients. The tumour suppressor function of WTX was further confirmed by observation of its ability to prevent GC cell proliferation, migration and invasion in vitro and inhibit GC cell proliferation and metastasis in vivo*.* The results of these studies confirmed that WTX is a tumour suppressor gene in GC and that the loss of WTX is at least partially responsible for GC progression.

The mechanism by which WTX functions as a GC tumour-suppressor was analysed for the first time in the present study. Both our gene expression microarray data (GSE114353) and public microarray data (GSE34715) revealed that WTX loss is associated with the aberrant activation of the PI3K/AKT/mTOR pathway, which induces the proliferation and metastasis of GC cells. The regulation of PI3K/AKT/mTOR pathway activity may be one of important mechanisms by which WTX regulates GC progression. The PI3K/AKT/mTOR signaling pathway is known to be frequently activated in GC and plays a crucial role in mediating multiple cellular functions, including cell proliferation, metastasis, and resistance to chemotherapy [24, 36–39]. PI3K/AKT/mTOR functions as an important pathway to regulate the progression of GC [40–43]. The Western blot and IHC staining results further confirmed that WTX negatively regulates PI3K/AKT/mTOR pathway activity and inhibits GC proliferation. For the first time, the results of the present study demonstrated that WTX regulates GC progression by preventing PI3K phosphorylation to inhibit PI3K/AKT/mTOR pathway activation. Our results indicated that the loss of WTX may be one of the causes leading to aberrant PI3K/AKT/mTOR signaling pathway activation in GC, which promotes the progression and metastasis of GC.

Aberrant miRNAs regulation is one of the key reasons leading to target gene silencing. To elucidate the mechanism driving WTX loss, we performed miRNA array analyses of GC and matched normal gastric mucous tissues with low and high WTX expression, respectively, resulting in the identification of miR-20a as a candidate regulator of WTX expression. Subsequently, we performed a bioinformatics analysis based on public miRNA array data and identified miR-20a-5p as a candidate regulator of WTX. It was previously reported that miR-20a is highly expressed in hepatocellular carcinoma [44], lung cancer [45], colon cancer [46], and other tumours [47, 48], and functions as an oncogene. Furthermore, miR-20a can be used as a biomarker in the diagnosis and prognosis of GC [49, 50]. The results of the above studies and reports suggests that miR-20a-5p is the most likely candidate capable of regulating WTX expression and GC progression. However, the relationship between miR-20a with WTX in GC has remained uninvestigated. To assess the potential miR-20a-mediated regulation of WTX and the associated mechanism, we performed a luciferase reporter assay and confirmed that WTX is a target of miR-20a-5p. Subsequently, the results of a miR-20a-5p functional analysis showed that miR-20a-5p can inhibit WTX expression and promote GC cell proliferation, invasion, and metastasis both in vitro and in vivo. Thus, the results of our study demonstrated that the aberrant upregulation of miR-20a-5p promotes GC progression by inducing WTX loss. In all GC cell line and public TCGA and GEO data assayed, all KEGG pathway analyses and verification experiments confirmed that miR-20a-5p positively regulates PI3K/AKT pathway activation by inhibiting WTX expression, thereby promoting GC progression.

## Conclusion

As a tumour suppressor gene, WTX loss is associated with GC cell metastasis and poor survival of GC patients. Under normal conditions, WTX inhibits PI3K/AKT/mTOR pathway activity by inhibiting PI3K phosphorylation. During GC progression, this biological process could be reversed by aberrantly high miR-20a-5p expression, which inhibits WTX expression and induces PI3K phosphorylation, thereby activating the PI3K/AKT/mTOR pathway and promoting cell proliferation and migration. The results of the present study elucidated a new molecular mechanism in which miR-20a-5p promotes GC development by regulating WTX expression to control PI3K/AKT/mTOR pathway activity. These findings provide a new understanding of GC progression to guide clinical practices.

## Supplementary information

**Additional file 1: Fig. S1.** WTX negatively regulates GC migration and proliferation. **a**-**b** Western blot analysis of WTX expression in the indicated cell lines. GAPDH is shown as a loading control. **c**-**d** Transwell migration analyses of the indicated cell lines. means±SD, *n* = 3. Two-tailed Student’s *t*-test. **e**-**h** Wound-healing analyses of the migration of the indicated cell lines. means±SD, *n* = 3. Two-tailed Student’s *t*-test. **i** Images of subcutaneous tumours formed by the indicated cell lines. *n* = 3. **j** Growth curves of subcutaneous tumours formed by the indicated cell lines. *P < 0.05, means±SD, *n* = 7. Two-tailed Student’s *t*-test. **k** Weight analysis of subcutaneous tumours formed by the indicated cell lines. ***P < 0.001, means±SD, *n =* 7. Two-tailed Student’s *t*-test.

**Additional file 2: Fig. S2.** WTX inhibits PI3K/AKT/mTOR pathway activation. **a** The top 20 hits from the KEGG pathway enrichment analysis identified using AGS. W & AGS.veh microarray data. **b**-**c** The top 20 hits from the KEGG pathway enrichment analysis performed with GSE34715.

**Additional file 3: Fig. S3.** MiR-20a-5p is upregulated in GC. **a** Based on analysis of the GSE94882 dataset, the ratio (N/C) of miRNA expression in 5 pairs of GC (C) and adjacent normal mucosal tissues (N). **b** Volcano plot of differentially expressed miRNAs (DEMs) in the GSE94882 dataset. DEMs with *Log2 Fold Change* > 1 and *P* < 0.05 were labelled red; DEMs with *Log2 Fold Change* < − 1 and *P* < 0.05 were labelled green. **c** Predicted miR-20a-5p target sequence in WTX-3’UTR (Wt) and a mutant containing 3 mutated nucleotides in the seed sequence of miR-20a-5p (Mut). **d** RT-PCR analyses of miR-20a-5p expression in GC cell lines. ****P < 0.0001, ***P < 0.001, **P < 0.01, *P < 0.05, means±SD, *n* = 3. Two-tailed Student’s *t*-test. **e** MiRNA expression heatmap of GC and adjacent normal mucosal tissues based on the GSE93415 dataset. T: Tumour, Healthy: H. *n* = 20. **f** Volcano plot of DEMs in GC and adjacent normal mucosal tissues based on the GSE93415 dataset. DEMs with *Log2 Fold Change* > 1 and *P* < 0.05 are labelled red; DEMs with *Log2 Fold Change* < − 1 and *P* < 0.05 are labelled green. **g** MiR-20a-5p expression in the GSE23739 dataset. means±SD, *n* = 40. Two-tailed Student’s *t*-test. h MiR-20a-5p expression in GC and matched normal tissues based on TCGA. means±SD, *n* = 41. Two-tailed Student’s *t*-test.

**Additional file 4: Fig. S4.** MiR-20a-5p regulates GC proliferation and migration. **a**-**b** RT-PCR analysis of miR-20a-5p expression in the indicated cell lines. ****P < 0.0001, ***P < 0.001, means±SD, *n* = 3. Two-tailed Student’s *t*-test. **c**-**d** Representative images from wound-healing assays of the indicated cell lines. **e** Statistical analysis of ISH staining scores of miR-20a-5p expression in subcutaneous tumours formed by the indicated cells. means±SD, *n* = 7. Two-tailed Student’s *t*-test. **f**-**h** Statistical analysis of IHC staining scores for WTX, p-AKT, and p-mTOR expression in subcutaneous tumours formed by the indicated cells. means±SD, *n* = 7. Two-tailed Student’s *t*-test. **i**-**j** The percentage of cells staining positive for Ki-67 in subcutaneous tumours. means±SD, *n* = 7. Two-tailed Student’s *t*-test.

**Additional file 5: Figure S5.** Mutual downstream pathway of WTX and miR-20a-5p. **a** Data processing scheme of gene expression profiling for GC obtained from TCGA. **b** Volcano plot of DEGs in GC with high miR-20a-5p expression and matched normal tissues obtained from TCGA. DEGs with |*Log2 Fold Change|* > 1 and *P* < 0.01 are labelled red, while other DEGs are labelled black. **c** A heatmap based on 46 overlapping genes of the PI3K/AKT signaling pathway in GC with high miR-20a-5p expression and matched normal tissues obtained from TCGA.

## Data Availability

The raw data for the mRNA and mircoRNA arrays have been deposited in the Gene Expression Omnibus under the accession code GSE114353 (https://www.ncbi.nlm.nih.gov/geo/query/acc.cgi?acc=GSE114353) and GSE94882 (https://www.ncbi.nlm.nih.gov/geo/query/acc.cgi?acc=GSE94882).

## Abbreviations

AKT: Serine/threonine-specific protein kinase
CCK8: Cell counting kit-8
GEO: Gene Expression Omnibus
IF: Immunofluorescence
IHC: Immunohistochemistry
mTOR: Mammalian target of rapamycin
PBS: Phosphate-buffered saline
PI3K: Phosphatidylinositol-3 kinase
WTX: Wilms tumour gene on the X chromosome
