# Correction to: Mir-20a-5p induced WTX deficiency promotes gastric cancer progressions through regulating PI3K/AKT signaling pathway

**DOI:** 10.1186/s13046-020-01809-2

**Published:** 2021-01-28

**Authors:** Jian Li, Danli Ye, Peng Shen, Xiaorong Liu, Peirong Zhou, Guifang Zhu, Yangwei Xu, Yun Fu, Xuanqi Li, Jingbo Sun, Jia Xu, Qingling Zhang

**Affiliations:** 1 Department of Pathology, Guangdong Provincial People’s Hospital, Guangdong Academy of Medical Sciences, Guangzhou, 510000 Guangdong Province People’s Republic of China; 2 Department of Pathology, School of Basic Medical Science, Southern Medical University, Guangzhou, Guangdong Province 510282 People’s Republic of China; 3 Department of Pathology, School of Basic Medical Sciences, Xinxiang Medical University, Xinxiang, 453003 Henan Province People’s Republic of China; 4 Department of Oncology, Nanfang Hospital, Southern Medical University, Guangzhou, 510282 Guangdong Province People’s Republic of China; 5 Department of Oncological Sciences, The Tisch Cancer Institute, Icahn School of Medicine at Mount Sinai, New York, NY 10029 USA

**Correction to: J Exp Clin Cancer Res 39, 212 (2020)**

**https://doi.org/10.1186/s13046-020-01718-4**

Following publication of the original article [1], the authors reported that author affiliation 1 needs to be revised, because the author’s hospital official requests the author affiliation must conform to the hospital’s standard. In addition, another author affiliation needs to be added for the authors Jian Li and Yun Fu.

The updated author affiliations should be:

Jian Li1,2,3†, Danli Ye1,2†, Peng Shen4†, Xiaorong Liu1,2, Peirong Zhou1,2, Guifang Zhu1,2, Yangwei Xu1,2, Yun Fu1,2,3, Xuanqi Li 1, Jingbo Sun1,2, Jia Xu5, Qingling Zhang1*

1Department of Pathology, Guangdong Provincial People’s Hospital, Guangdong Academy of Medical Sciences, Guangzhou 510000, Guangdong Province, People’s Republic of China.

2Department of Pathology, School of Basic Medical Science, Southern Medical University, Guangzhou 510282, Guangdong Province, People’s Republic of China.

3Department of Pathology, School of Basic Medical Sciences, Xinxiang Medical University, Xinxiang 453003, Henan Province, People’s Republic of China.

4Department of Oncology, Nanfang Hospital, Southern Medical University, Guangzhou 510282, Guangdong Province, People’s Republic of China.

5Department of Oncological Sciences, The Tisch Cancer Institute, Icahn School of Medicine at Mount Sinai, New York, NY 10029, USA.

The original article has been updated.
