# Supplementary figures and images for: Mir-20a-5p induced WTX deficiency promotes gastric cancer progressions through regulating PI3K/AKT signaling pathway
Source: J Exp Clin Cancer Res. 2020 Oct 8;39:212. doi: 10.1186/s13046-020-01718-4 (PMC7545863; doi:10.1186/s13046-020-01718-4)

A

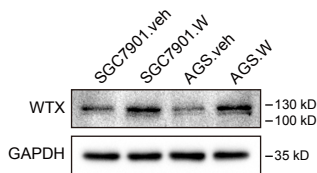

B

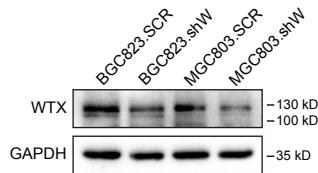

C

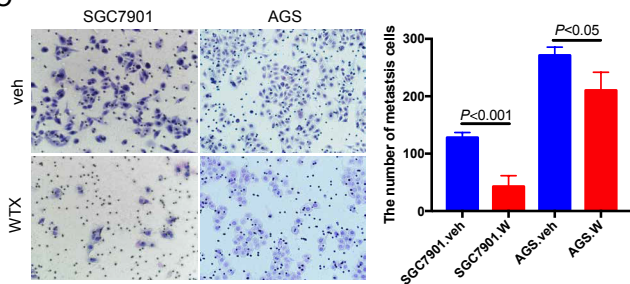

D

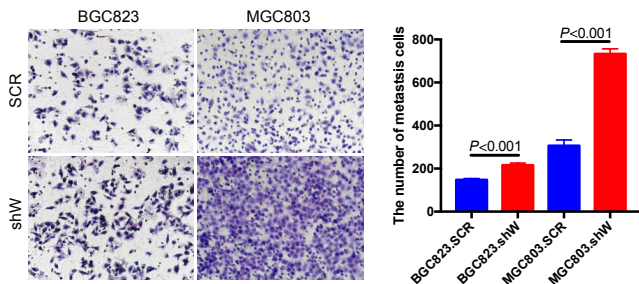

E

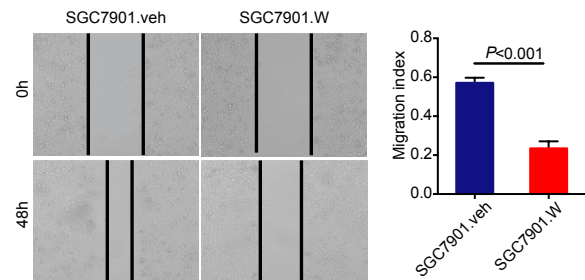

F

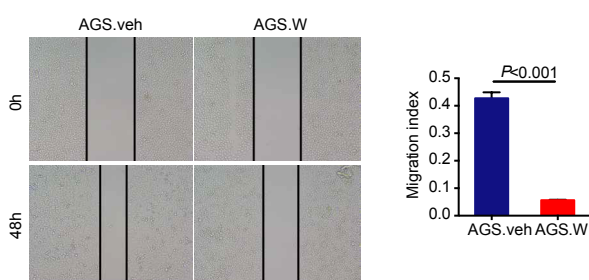

G

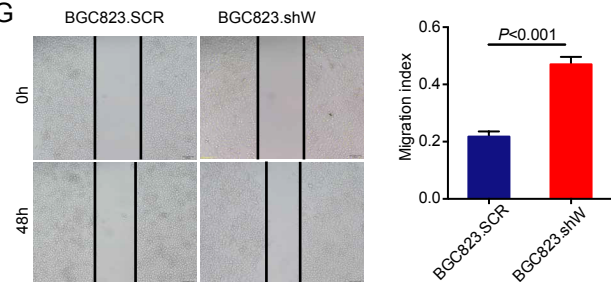

H

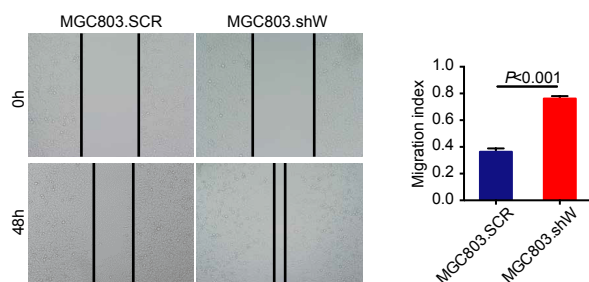

I

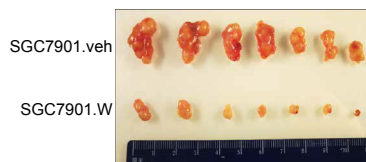

J

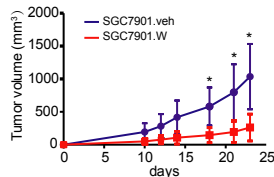

K

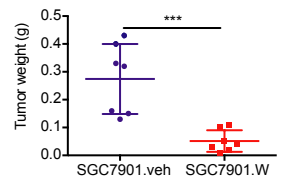

Supplement: Supplementary file 1 — Additional file 1: Fig. S1. WTX negatively regulates GC migration and proliferation. a-b Western blot analysis of WTX expression in the indicated cell lines. GAPDH is shown as a loading control. c-d Transwell migration analyses of the indicated cell lines. means±SD, n = 3. Two-tailed Student’s t-test. e-h Wound-healing analyses of the migration of the indicated cell lines. means±SD, n = 3. Two-tailed Student’s t-test. i Images of subcutaneous tumours formed by the indicated cell lines. n = 3. j Growth curves of subcutaneous tumours formed by the indicated cell lines. *P < 0.05, means±SD, n = 7. Two-tailed Student’s t-test. k Weight analysis of subcutaneous tumours formed by the indicated cell lines. ***P < 0.001, means±SD, n = 7. Two-tailed Student’s t-test. [file 13046_2020_1718_MOESM1_ESM.pdf]

A

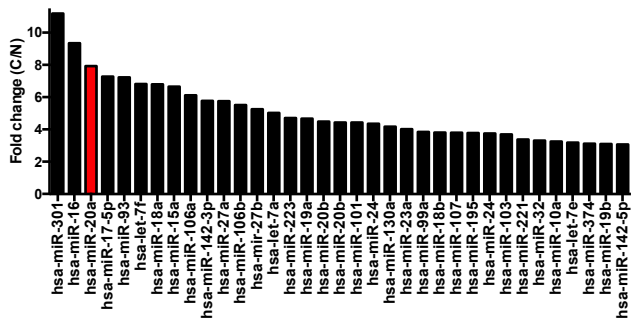

C

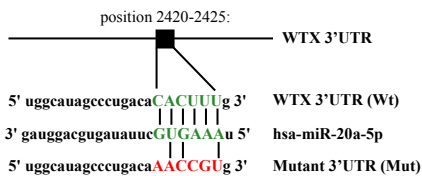

E

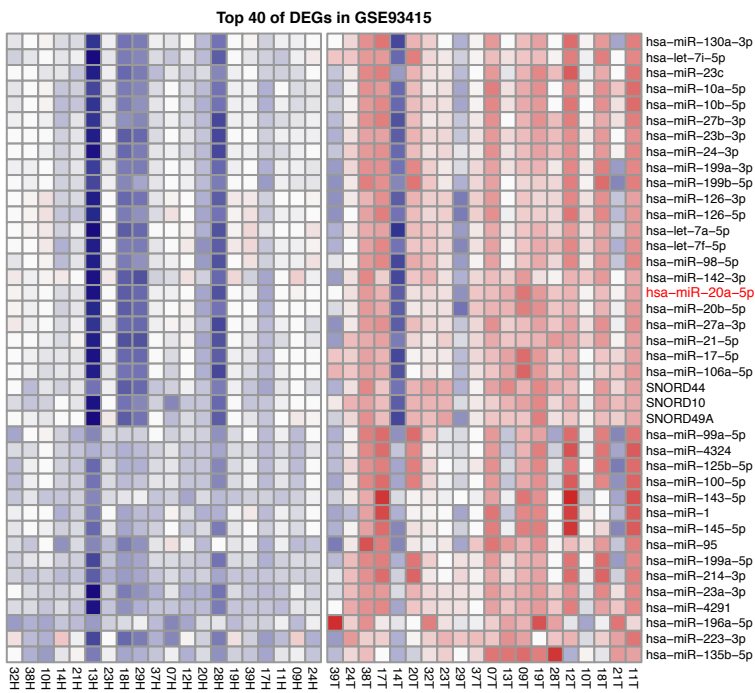

B

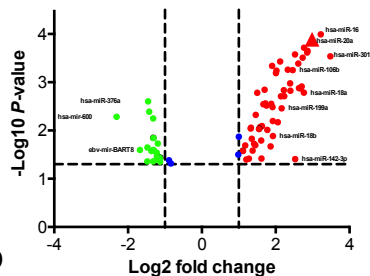

D

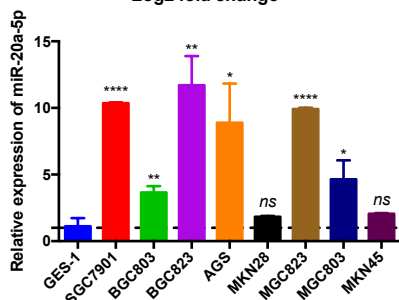

F

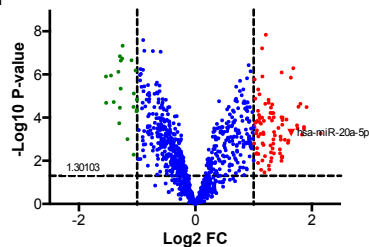

G

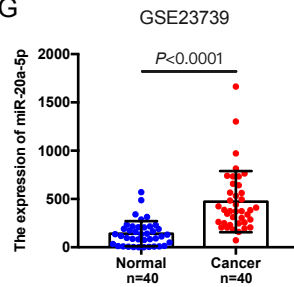

H

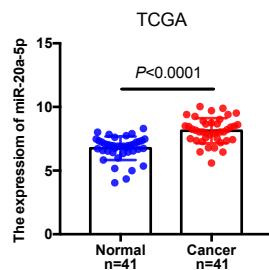

Supplement: Supplementary file 3 — Additional file 3: Fig. S3. MiR-20a-5p is upregulated in GC. a Based on analysis of the GSE94882 dataset, the ratio (N/C) of miRNA expression in 5 pairs of GC (C) and adjacent normal mucosal tissues (N). b Volcano plot of differentially expressed miRNAs (DEMs) in the GSE94882 dataset. DEMs with Log2 Fold Change > 1 and P < 0.05 were labelled red; DEMs with Log2 Fold Change < − 1 and P < 0.05 were labelled green. c Predicted miR-20a-5p target sequence in WTX-3’UTR (Wt) and a mutant containing 3 mutated nucleotides in the seed sequence of miR-20a-5p (Mut). d RT-PCR analyses of miR-20a-5p expression in GC cell lines. ****P < 0.0001, ***P < 0.001, **P < 0.01, *P < 0.05, means±SD, n = 3. Two-tailed Student’s t-test. e MiRNA expression heatmap of GC and adjacent normal mucosal tissues based on the GSE93415 dataset. T: Tumour, Healthy: H. n = 20. f Volcano plot of DEMs in GC and adjacent normal mucosal tissues based on the GSE93415 dataset. DEMs with Log2 Fold Change > 1 and P < 0.05 are labelled red; DEMs with Log2 Fold Change < − 1 and P < 0.05 are labelled green. g MiR-20a-5p expression in the GSE23739 dataset. means±SD, n = 40. Two-tailed Student’s t-test. h MiR-20a-5p expression in GC and matched normal tissues based on TCGA. means±SD, n = 41. Two-tailed Student’s t-test. [file 13046_2020_1718_MOESM3_ESM.pdf]

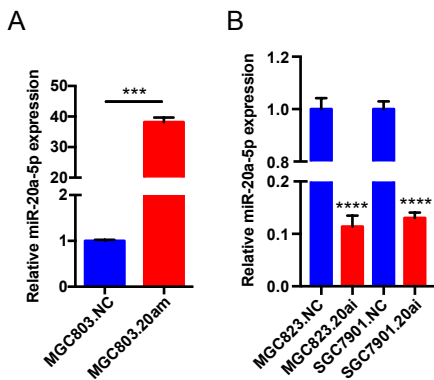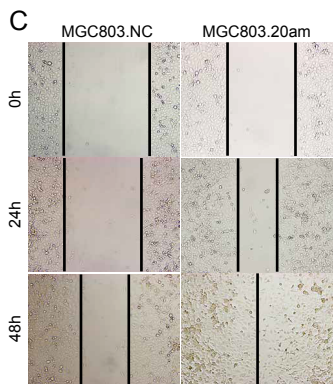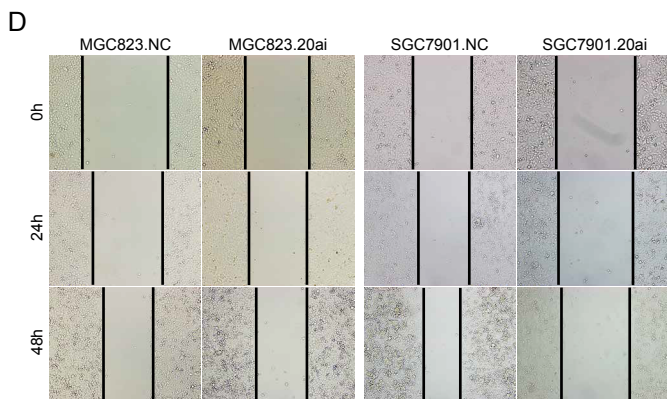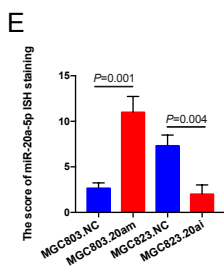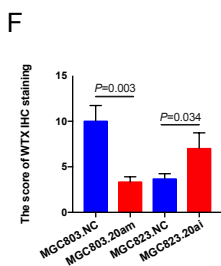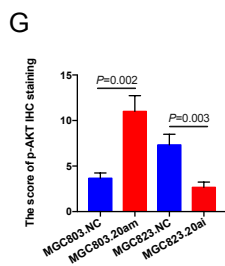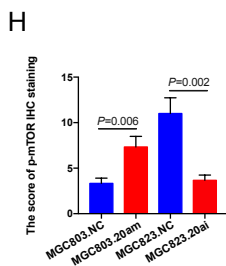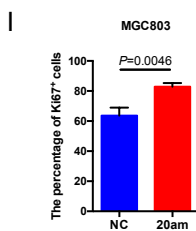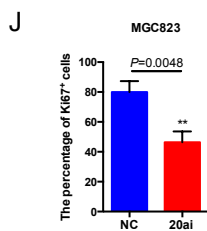

Supplement: Supplementary file 4 — Additional file 4: Fig. S4. MiR-20a-5p regulates GC proliferation and migration. a-b RT-PCR analysis of miR-20a-5p expression in the indicated cell lines. ****P < 0.0001, ***P < 0.001, means±SD, n = 3. Two-tailed Student’s t-test. c-d Representative images from wound-healing assays of the indicated cell lines. e Statistical analysis of ISH staining scores of miR-20a-5p expression in subcutaneous tumours formed by the indicated cells. means±SD, n = 7. Two-tailed Student’s t-test. f-h Statistical analysis of IHC staining scores for WTX, p-AKT, and p-mTOR expression in subcutaneous tumours formed by the indicated cells. means±SD, n = 7. Two-tailed Student’s t-test. i-j The percentage of cells staining positive for Ki-67 in subcutaneous tumours. means±SD, n = 7. Two-tailed Student’s t-test. [file 13046_2020_1718_MOESM4_ESM.pdf]

A

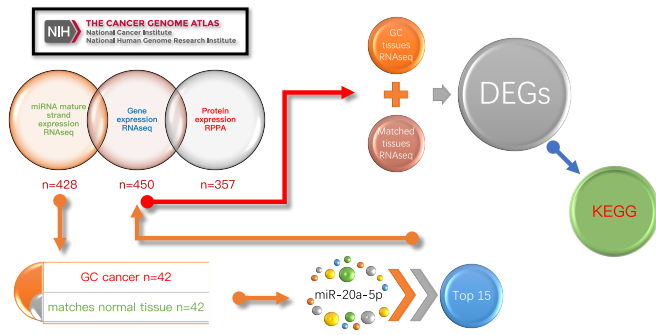

B

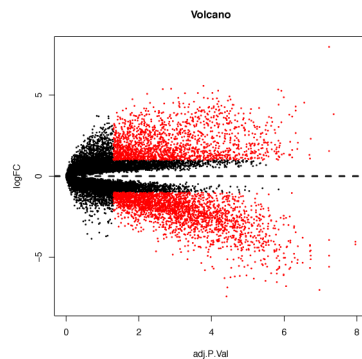

C

DEGs in PI3K-Akt signaling pathway

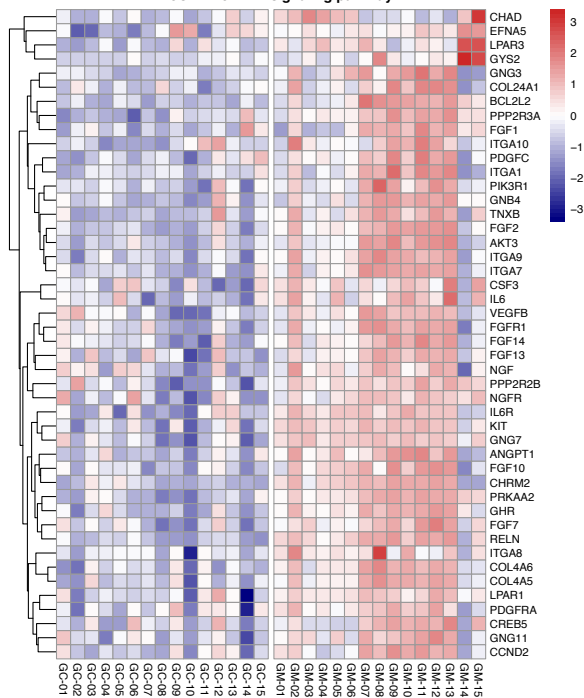

Supplement: Supplementary file 5 — Additional file 5: Figure S5. Mutual downstream pathway of WTX and miR-20a-5p. a Data processing scheme of gene expression profiling for GC obtained from TCGA. b Volcano plot of DEGs in GC with high miR-20a-5p expression and matched normal tissues obtained from TCGA. DEGs with |Log2 Fold Change| > 1 and P < 0.01 are labelled red, while other DEGs are labelled black. c A heatmap based on 46 overlapping genes of the PI3K/AKT signaling pathway in GC with high miR-20a-5p expression and matched normal tissues obtained from TCGA. [file 13046_2020_1718_MOESM5_ESM.pdf]
