# Supplementary material for: Mir-20a-5p induced WTX deficiency promotes gastric cancer progressions through regulating PI3K/AKT signaling pathway
Source: J Exp Clin Cancer Res. 2020 Oct 8;39:212. doi: 10.1186/s13046-020-01718-4 (PMC7545863; doi:10.1186/s13046-020-01718-4)
Supplement: Supplementary file 2 — Additional file 2: Fig. S2. WTX inhibits PI3K/AKT/mTOR pathway activation. a The top 20 hits from the KEGG pathway enrichment analysis identified using AGS. W & AGS.veh microarray data. b-c The top 20 hits from the KEGG pathway enrichment analysis performed with GSE34715. [file 13046_2020_1718_MOESM2_ESM.pdf]

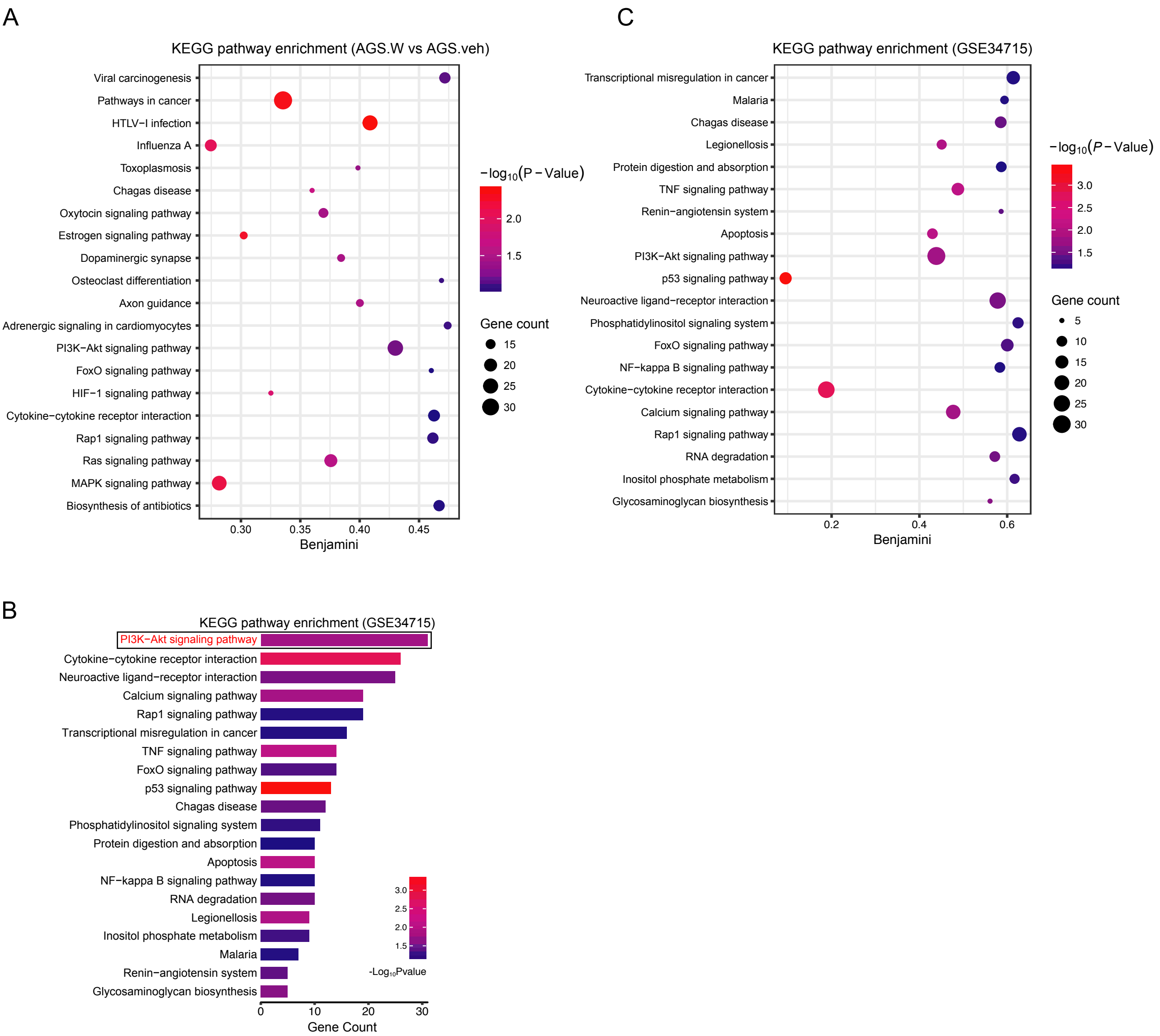
A
KEGG pathway enrichment (AGS.W vs AGS.veh)
Viral carcinogenesis
Pathways in cancer
HTLV−I infection
Influenza A
Toxoplasmosis
Chagas disease
Oxytocin signaling pathway
Estrogen signaling pathway
Dopaminergic synapse
Osteoclast differentiation
Axon guidance
Adrenergic signaling in cardiomyocytes
PI3K−Akt signaling pathway
FoxO signaling pathway
HIF−1 signaling pathway
Cytokine−cytokine receptor interaction
Rap1 signaling pathway
Ras signaling pathway
MAPK signaling pathway
Biosynthesis of antibiotics
0.30
0.35
0.40
0.45
Benjamini
−log10(P − Value)
2.0
1.5
Gene count
15
20
25
30
C
KEGG pathway enrichment (GSE34715)
Transcriptional misregulation in cancer
Malaria
Chagas disease
Legionellosis
Protein digestion and absorption
TNF signaling pathway
Renin−angiotensin system
Apoptosis
PI3K−Akt signaling pathway
p53 signaling pathway
Neuroactive ligand−receptor interaction
Phosphatidylinositol signaling system
FoxO signaling pathway
NF−kappa B signaling pathway
Cytokine−cytokine receptor interaction
Calcium signaling pathway
Rap1 signaling pathway
RNA degradation
Inositol phosphate metabolism
Glycosaminoglycan biosynthesis
0.2
0.4
0.6
Benjamini
−log10(P − Value)
3.0
2.5
2.0
1.5
Gene count
5
10
15
20
25
30
B
KEGG pathway enrichment (GSE34715)
PI3K−Akt signaling pathway
Cytokine−cytokine receptor interaction
Neuroactive ligand−receptor interaction
Calcium signaling pathway
Rap1 signaling pathway
Transcriptional misregulation in cancer
TNF signaling pathway
FoxO signaling pathway
p53 signaling pathway
Chagas disease
Phosphatidylinositol signaling system
Protein digestion and absorption
Apoptosis
NF−kappa B signaling pathway
RNA degradation
Legionellosis
Inositol phosphate metabolism
Malaria
Renin−angiotensin system
Glycosaminoglycan biosynthesis
3.0
2.5
2.0
1.5
-Log10Pvalue
0
10
20
30
Gene Count
